# Prevalence, associated risk factors and satellite imagery analysis in predicting soil-transmitted helminth infection in Nakhon Si Thammarat Province, Thailand

**DOI:** 10.1038/s41598-025-14221-7

**Published:** 2025-08-04

**Authors:** Jarawadee Muenjak, Jutarat Thongrod, Chanakan Choodamdee, Pongphan Pongpanitanont, Manachai Yingklang, Tongjit Thanchomnang, Sakhone Laymanivong, Penchom Janwan

**Affiliations:** 1 Department of Medical Technology, School of Allied Health Sciences, Walailak University, Nakhon Si Thammarat, 80160 Thailand; 2 Health Sciences (International Program), College of Graduate Studies, Walailak University, Nakhon Si Thammarat, 80160 Thailand; 3 Faculty of Public Health, Burapha University, Chonburi, 20131 Thailand; 4 Faculty of Medicine, Mahasarakham University, Maha Sarakham, 44000 Thailand; 5 Centre of Malariology, Parasitology and Entomology, Ministry of Health, P.O. Box 0100, Vientiane, Laos; 6 Hematology and Transfusion Science Research Center, Walailak University, Nakhon Si Thammarat, 80160 Thailand

**Keywords:** Soil-transmitted helminth, Prevalence, Risk factor, Satellite imagery analysis, Predictive modeling, Thailand, Risk factors, Infectious diseases, Applied mathematics, Statistics

## Abstract

Soil-transmitted helminth (STH) infections remain a significant public health concern in rural areas, often leading to nutritional and physical impairment, particularly in children. This study aimed to assess the prevalence and associated factors of STH infections among schoolchildren in Thasala District, Nakhon Si Thammarat Province, Thailand, and to develop a predictive model for identifying high-risk areas using satellite imagery data. A cross-sectional study was conducted with 319 primary schoolchildren from six sub-districts in Thasala District. Stool samples were analyzed for STH infections using the formalin ethyl acetate concentration technique (FECT) and agar plate culture (APC), while behavioral data were collected through questionnaires to identify key risk factors. We developed an innovative predictive model by integrating convolutional neural networks (CNNs) for land-use classification of satellite imagery with artificial neural networks (ANNs) following dimensionality reduction through principal component analysis (PCA). The STH infections were detected in 31 samples (9.72%), with higher prevalence in males (11.38%) than females (8.67%). Mono-infections predominated, with *Trichuris trichiura* (5.02%) and hookworm (3.49%) being the most frequent. Mixed infections accounted for 1.25%, primarily co-infections of hookworm with *T. trichiura* (0.94%) or *Strongyloides stercoralis* (0.31%). Not cutting nails was identified as a significant behavioral factor associated with STH infections (*p* = 0.047), while other behavioral factors showed no statistical significance. From the satellite imagery analysis, specific environmental features, particularly higher proportions of agricultural land and closer proximity to water bodies, were positively associated with elevated STH prevalence. The modelling approach generated spatial risk maps for STH infections, providing a cost-effective tool for identifying high-risk transmission zones. These findings highlight that STH infections persist among rural Thai schoolchildren, with poor hygiene practices as a contributing factor. Strengthening hygiene education, improving sanitation, and implementing targeted environmental interventions are essential for effective control.

**Supplementary Information:**

The online version contains supplementary material available at 10.1038/s41598-025-14221-7.

## Introduction

Soil-transmitted helminth infections represent the most prevalent neglected tropical diseases globally, imposing a significant disease burden in low- and middle-income countries, particularly among school-aged children^1,2^. The predominant STHs include *Ascaris lumbricoides*, *Trichuris trichiura*, hookworm, and *Strongyloides stercoralis*^3,4^. These infections, associated with poor hygiene, contaminated food or water consumption, and low socioeconomic status^4,5^. The World Health Organization estimates that approximately 1.5 billion people are infected with STHs^4^. The highest prevalence of STH infections occurs in sub-Saharan Africa, the Americas, South Asia, and Southeast Asia^4,6,7^. In Thailand, despite decreasing trends in STH infection rates resulting from disease surveillance and control policies outlined in the Thai Ministry of Public Health roadmap 2017–2026, these infections continue to persist across various regions of the country^8,9^. The nationwide helminthiases survey conducted in Thailand in 2019 across all age groups revealed an overall prevalence of helminthic infections of 9.79%, with over 14 species identified. Hookworms demonstrated the highest prevalence (4.47%). The southern region of Thailand reported the highest hookworm infection rates, ranging from 8.06 to 23.08% across the ten southern provinces. This nationwide study provided geographical distribution at the provincial level and generated predicted prevalence maps for hookworms using an inverse distance weighting spatial interpolation technique^8^. While predicted prevalence maps of STH infections are essential tools for guiding public health interventions, their limitations necessitate careful interpretation and consideration of additional data and methodologies to enhance accuracy and applicability. Despite national-level mapping, detailed province-specific studies are crucial, as risk factors and transmission patterns may vary significantly between localities. Previous research in Nopphitam District, Nakhon Si Thammarat Province, southern Thailand, documented an overall STH prevalence of approximately 11.0% among primary schoolchildren, with hookworm predominating at 10.7%, followed by *T. trichiura* at 0.3%^10^. This investigation examined personal, environmental, and behavioral risk factors through a structured questionnaire, revealing that older age groups of primary schoolchildren (10–12 years) and inadequate handwashing before meals were significantly associated with hookworm infections. This study provided supporting spatial data characterizing the study area as having a distinct tropical hot and humid climate, which is suitable for the proliferation of STHs. However, there remains a critical gap in research regarding the development of risk area mapping for southern Thailand, particularly in relation to ongoing STH transmission. Geographic and environmental conditions significantly influence the spatial distribution of parasites. Advanced tools such as geographic information systems (GIS) and satellite imagery enable researchers to map high-risk areas and predict disease transmission patterns^8,11–13^. Recent studies have applied sophisticated machine learning methods, including random forest algorithms, ANNs, and CNNs, to enhance the analysis of environmental risk factors in remote or inaccessible regions. These approaches facilitate more effective parasite and/or vector surveillance and inform the development of targeted control strategies^11,14,15^.

Our study addresses a critical research gap by examining STH infection prevalence and risk factors among school-aged children in Thasala District, Nakhon Si Thammarat Province, southern Thailand. Our innovative approach integrates traditional parasitological methods with satellite imagery analysis to characterize diverse land-use patterns including agricultural, residential, forest, aquatic, and built environments. By correlating specific environmental contexts with infection data, we identify high-risk ecological niches facilitating parasite transmission. This methodology provides a cost-effective framework for targeted interventions in resource-limited settings without requiring extensive field surveys. Our findings enhance understanding of STH transmission dynamics in southern Thailand and offer a reproducible model for predicting infection hotspots applicable to similar endemic regions globally.

## Methods and materials

### Study area

**Fig. 1:**
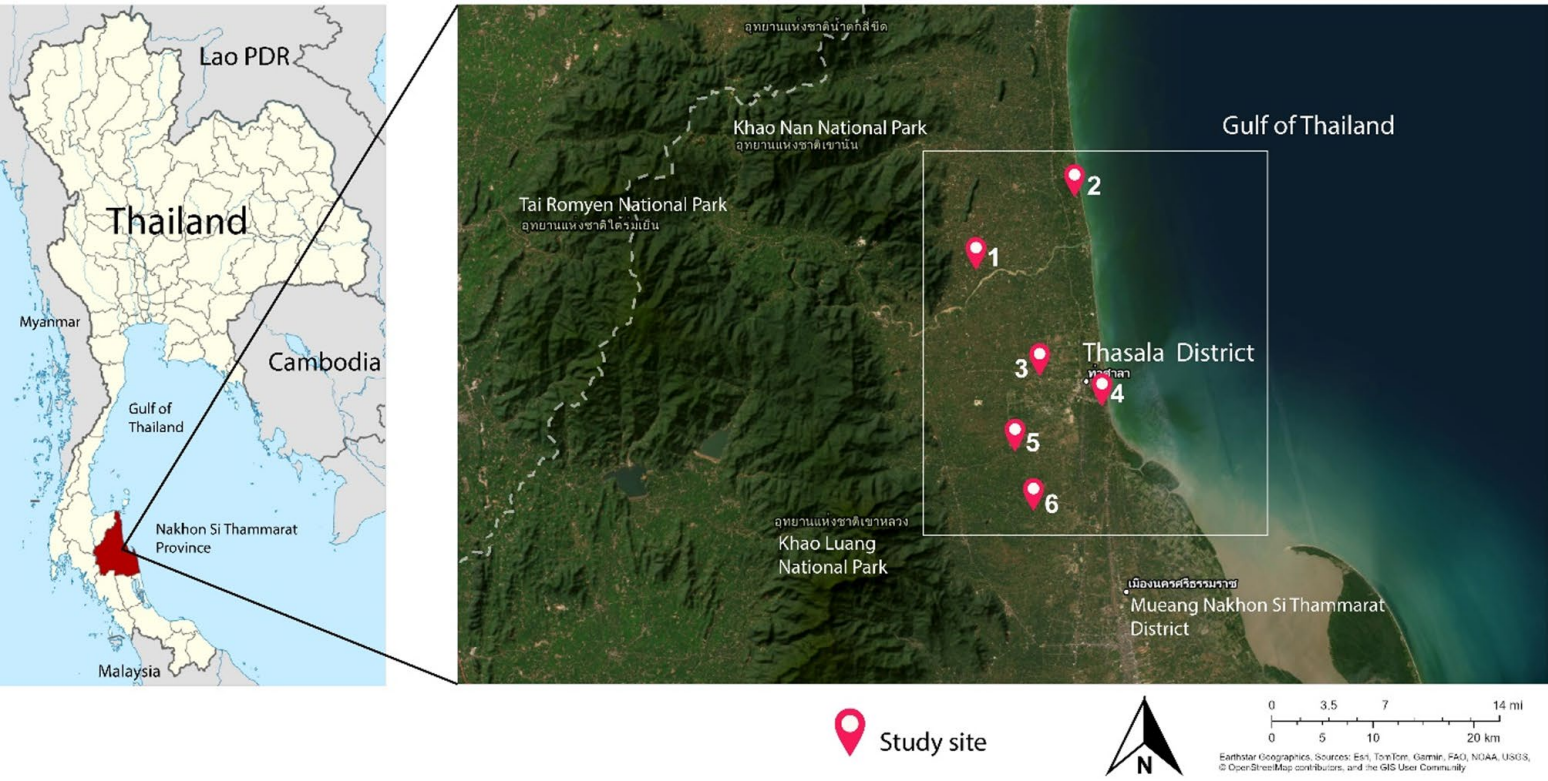
The study area, which includes six sub-districts in Thasala District, Nakhon Si Thammarat Province: (1) Taling Chan, (2) Klai, (3) Thai Buri, (4) Thasala, (5) Moklan, and (6) Don Tako. Map was modified from Wikipedia Commons: https://en.wikipedia.org/wiki/Tha_Sala_district and ArcGIS software by Esri. Sources: Esri, TomTom, Garmin, FAO, NOAA, USGS, OpenStreetMap contributors, and the GIS User Community. For more information about Esri software, please visit http://www.esri.com. All other layers were produced by the authors and are copyright-free.

A cross-sectional study was conducted from June to August 2024 in primary schools in Thasala District, Nakhon Si Thammarat Province, covering an area of approximately 375.53 square kilometers. Thasala is a coastal district along the Gulf of Thailand and is divided into 10 sub-districts. The topography consists of both sandy and clay soils, with most of the population engaged in agriculture^16^. In this study, six sub-districts were selected: Taling Chan, Klai, Thai Buri, Thasala, Moklan, and Don Tako. The selected areas were based on similar geographical characteristics: Klai and Thasala are adjacent to the sea; Taling Chan and Thai Buri are on similar longitudes with Klai and Thasala, respectively, so they can be compared; and Moklan and Don Tako are non-landlocked. This selection allowed for geographical diversity while ensuring that some sub-districts shared similar characteristics, making the study comprehensive and spatially balanced as show in Fig. 1.

### Sampling and sample size

A simple random sampling method from selected six sub-districts was used. Children, who had not received anthelminthics in the three months before attending the study, were included for screening of STHs infections. The sample size was calculated using the formula for estimating a finite population proportion^17^. Based on a prevalence rate (p) of 16%^10^, with a 95% confidence interval (z = 1.96) and a margin of error of 5% (d = 0.05), the estimated number of students aged 6–12 years was 10,711^18^. To allow for a drop-out rate of 25%, 254 individuals were required. However, a total of 319 participants had returned the assent and informed consent forms, so we included all individuals in the study.

### Stool sample collection and STHs detection

A clean, labeled stool collection container with a scoop attached inside was distributed to all students who consented to participate a few days prior to specimen collection. On the day of container distribution, we provided verbal instructions using age-appropriate language and supplied documentation with clear illustrated protocol suitable for their age group. Students were instructed to take the containers home and collect stool samples according to the provided guidelines. They were directed to collect approximately 5–6 g of their morning stool specimen using the provided scoop and place it in the container, avoiding contamination with urine, water, or soil. To ensure proper handling, parents or guardians were informed about the study through written consent forms and were requested to assist their children with the collection process when necessary, following the enclosed instructions. The students then returned the samples to their schools, where our staff collected and transported them to the Medical Technology Laboratory at Walailak University for intestinal parasite detection.

We employed two highly accurate laboratory techniques to maximize the detection of parasites: FECT and APC. The FECT is highly sensitive, capable of detecting minimal quantities of parasites, particularly helminth eggs and larvae. The APC method cultivates parasites on agar plates to observe nematode growth, thereby enhancing the sensitivity for detecting *S. stercoralis* and/or hookworm infections. The preparation of stool samples and parasite detection protocols were conducted as previously described^19,20^.

### Questionnaire survey

The questionnaire was administered by both the children and their parents, depending on the child’s ability to read and understand the questionnaire. For children who were unable to read, typically younger children in grades 1–3, a parent or guardian was instructed to complete the questionnaire on their behalf. This ensured that data collection remained accurate and comprehensive, regardless of the child’s ability to read. The questionnaire, adapted from previous research, was designed to evaluate various factors potentially associated with parasitic infections^10^. It included questions covering demographic information (gender and grade level), personal hygiene practices (handwashing, vegetable washing, and consumption of thoroughly cooked food), environmental behaviors (barefoot walking, toilet usage, and filtered water consumption), and risk behaviors (finger biting, interaction with pets, and nail trimming).

### Statistical analysis of STHs infections and risk factors

Demographic data were presented using percentages and participant counts. The prevalence of STHs infections calculated by the number of infected children divided by a total of sample collection at time point multiply one hundred. The association between STHs infections and related factors was examined using Fisher’s Exact Test. Statistical test was considered significant at a *p*-value of less than 0.05. All analyses were performed using STATA package version 10.1 (StataCorp LLC, College Station, TX, USA).

### Development of a predictive model to identify high-risk areas

**Fig. 2:**
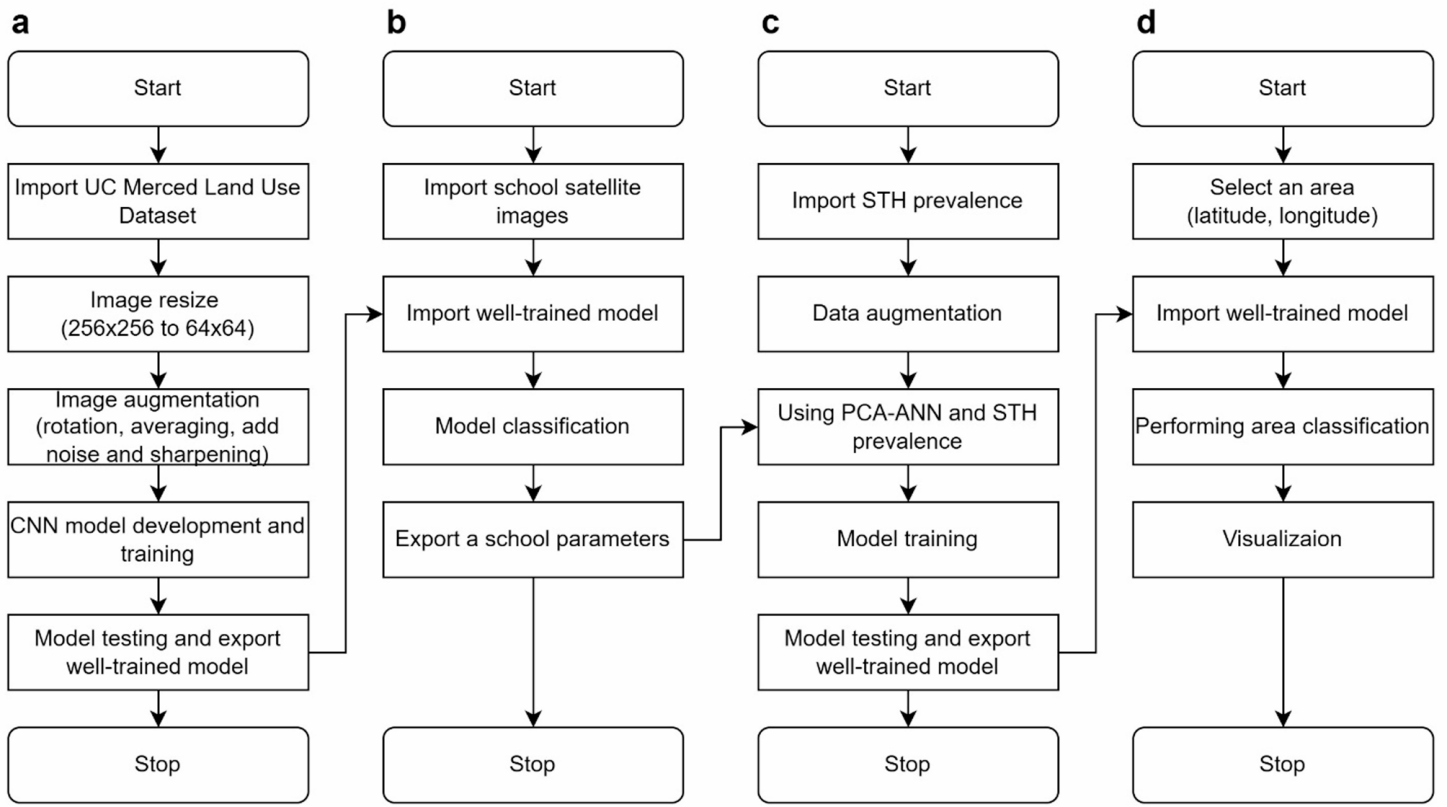
Four-stage satellite imagery analysis for STH transmission risk mapping. Our methodology includes: (**a**) CNN model development using UC Merced Land Use Dataset with image preprocessing; (**b**) Application of trained model to classify land-use patterns surrounding six schools in Thasala District; (**c**) Construction of a predictive model integrating parasitological diagnosis results with school environmental parameters; (**d**) Development of a geographic risk prediction system that generates spatial risk distribution visualizations based on coordinates.

**Fig. 3:**
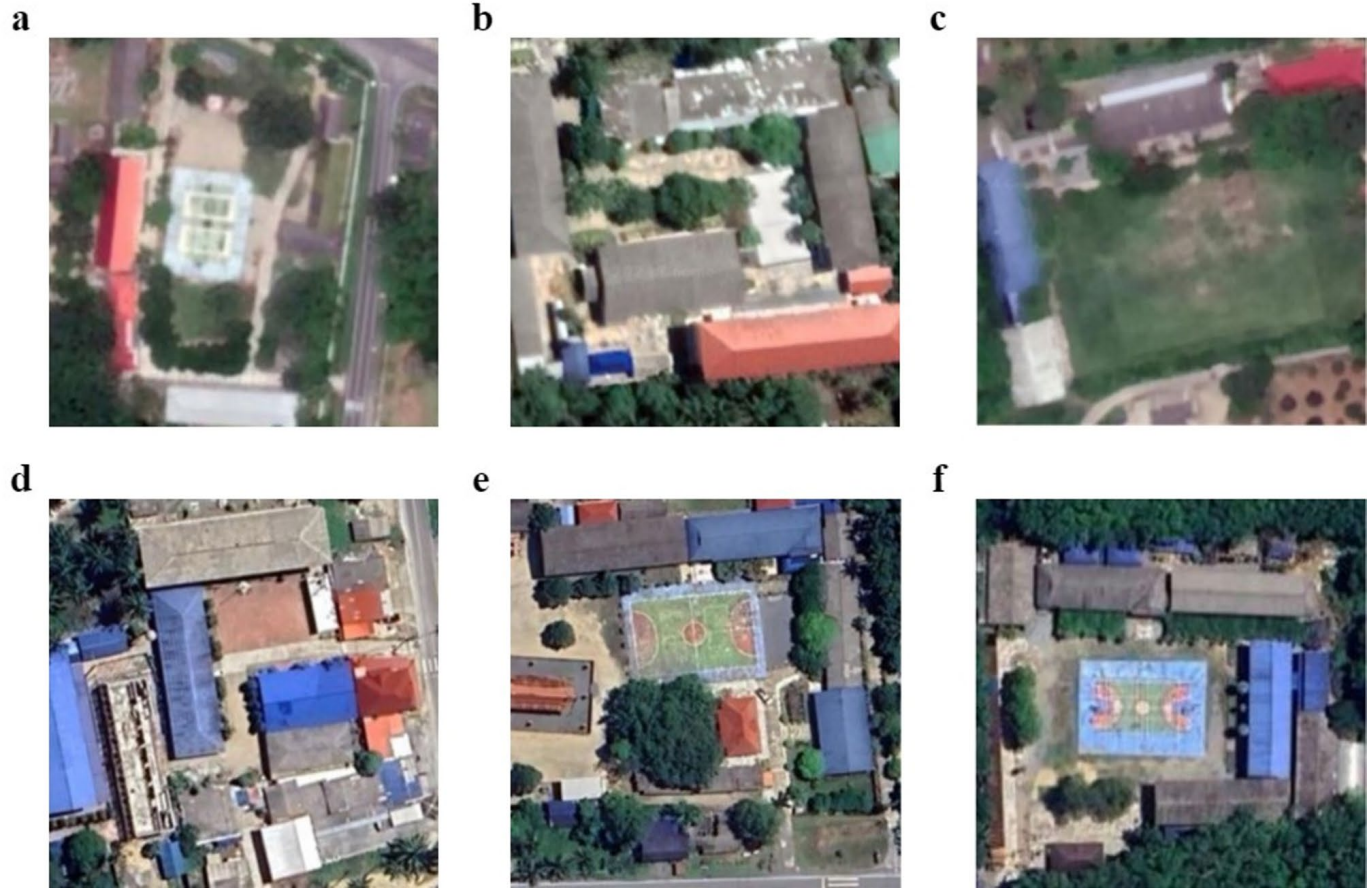
Representative satellite imagery of six school campuses across different sub-districts in Thasala District used for predictive STH infection risk modeling: (**a**) Thai Buri, (**b**) Thasala, (**c**) Taling Chan, (**d**) Klai, (**e**) Moklan, and (**f**) Don Tako sub-districts. The satellite imagery was obtained and visualized using Folium version 0.19.5 (https://python-visualization.github.io/folium/) in the Python programming environment.

Our study developed an innovative predictive model that integrates CNNs for land-use classification with ANNs that process PCA-refined data (Fig. 2). We employed CNNs for land-use classification using publicly available datasets from the USGS National Map Urban Area Imagery collection (21 classes, 100 images per class at 256 × 256 pixels)^21^. Model robustness was enhanced through image augmentation techniques including 90-degree rotations, averaging filters, noise introduction, and sharpening. We evaluated three CNN architectures^22^namely custom-designed CNN, ResNet50, and DenseNet121, using consistent hyperparameters and assessed their performance through accuracy, precision, recall, and F1-score metrics. The best-performing model was exported with optimized parameters to establish a foundation for the next step. Using a pre-validated classification model, we analyzed 30 satellite images from six schools (5 images per school) (Fig. 3), extracting unique architectural and locational parameters. This approach demonstrates how remote sensing and machine learning can enhance educational infrastructure analysis. All images were standardized to a resolution of 256 × 256 pixels to maintain uniformity in spatial resolution and facilitate comparative analyses across the dataset. To correlate satellite image parameters with STH prevalence, we integrated PCA with ANN. Our field survey-confirmed prevalence data and school parameters were combined and expanded using low variance noise. PCA reduced dimensionality (0.95 explained variance), with principal components feeding into an ANN for prevalence prediction. For area scanning, we established precise latitude-longitude boundaries with optimized step sizes to ensure comprehensive coverage. The well-trained CNN and ANN models worked collaboratively to classify regions, capturing both detailed features and broader patterns in satellite imagery. The resulting risk map was overlaid on original images, providing visual representation of spatial variations to support informed decision-making.

### Ethics declarations

The study’s protocol was approved by the Ethics Committee in Human Research Walailak University (Approval No: WUEC-24-177-01). The study followed the Declaration of Helsinki. The study’s purpose and procedures were explained to the participants prior to enrolment. Written informed consent was obtained from all participants and the parent or the legal guardian of the child before the study onset. All study participants infected with STHs were treated with mebendazole or ivermectin.

## Results

### Participant recruitment and prevalence of STHs infections

Stool samples and questionnaires were collected from children aged 6–12 years across six sub-districts: Taling Chan, Klai, Thai Buri, Thasala, Moklan, and Don Tako. A total of 917 children were randomly selected for participation, and 319 provided both stool samples and completed questionnaires, following assent and informed consent procedures. The number of participants from each sub-district is detailed in Table 1.

**Table 1:** Number of participants recruited and prevalence of STH infections by sub-district.

Sub-district	No. of randomly sampled students	No. consenting participants	% STH prevalence
Klai	153	44	6.82
			(Hw 2.27%, Hw + Tt 4.55%)
Thai Buri	96	49	6.12
			(Hw 4.08%, Tt 2.04%)
Thasala	310	59	32.20
			(Hw 5.08%, Tt 25.42%, Hw + Tt 1.70%)
Taling Chan	144	73	5.48
			(Hw 4.11%, Hw + Ss 1.37%)
Moklan	72	30	6.67
			(Hw 6.67%)
Don Tako	142	64	0.00
Total	917	319	

STHs: soil-transmitted helminths; Hw: hookworm; Tt: *T. trichiura*; Ss: *S. stercoralis*.

Among the 319 samples analyzed using the FECT and APC methods, 31 cases of SIHs infections were detected, representing an overall prevalence of 9.72%. Mono-infections were more common than mixed infections, with *T. trichiura* being the most frequently detected parasite (5.02%), followed by hookworm (3.49%). Mixed infections were less prevalent, accounting for 1.25% of cases. The most frequently observed co-infection was hookworm and *T. trichiura* (0.94%), followed by hookworm and *S. stercoralis* (0.31%) (Table 2).

**Table 2:** Frequency of parasitic infections using FECT and APC methods (*n* = 319).

Type of STHs infections	No. of positive STHs	% Positive	No. of FECT positive	No. of APC positive
**Mono-infection**				
*T. trichiura*	16	5.02	16	0
Hookworm	11	3.49	10	7
**Mixed infections**				
Hookworm & *T. trichiura*	3	0.94	3 (Hw & Tt eggs)	2 (Hw larva)
Hookworm & *S. stercoralis*	1	0.31	1 (Hw egg)	1 (Ss larva)
**All**	31	9.72		

STHs: soil-transmitted helminths; Hw: hookworm; Tt: *T. trichiura*; Ss: *S. stercoralis*.

### Association between STHs infections and risk factors

An analysis of 319 schoolchildren revealed no significant associations between STH infections and gender, grade level, handwashing practices, food hygiene, walking barefoot, defecation practices, or drinking water source (*p* > 0.05). However, not keeping nails short was significantly associated with infection (16.18% vs. 7.97%, *p* = 0.047). Playing with pets was also associated with a higher infection (13.82% vs. 7.14%), nearing statistical significance (*p* = 0.05) (Table 3).

**Table 3:** Analysis of the association between STHs infections and related factors (*n* = 319).

Variables	All (*n* = 319)	No. of positive STHs (%)		*p*-value*	
		Yes	No		
**Gender**					
Female	196	17 (8.67)	179 (91.33)		
Male	123	14 (11.38)	109 (88.62)	0.43	
**Grade**					
1–3	148	12 (8.11)	136 (91.89)		
4–6	171	19 (11.11)	152 (88.89)	0.45	
**Washing hands before eating**					
No	75	6 (8.00)	69 (92.00)		
Yes	244	25 (10.25)	219 (89.75)	0.66	
**Washing hands after using the toilet**					
No	36	4 (11.11)	32 (88.89)		
Yes	283	27 (9.54)	256 (90.46)	0.77	
**Washing fresh vegetables before consuming them**					
No	7	1 (14.29)	6 (85.71)		
Yes	312	30 (9.62)	282 (90.38)	0.52	
**Only eat fully cooked food**					
No	30	3 (10.00)	27 (90.00)		
Yes	289	28 (9.69)	261 (90.31)	0.57	
**Walking barefoot on the ground**					
No	223	22 (9.87)	201 (90.13)		
Yes	96	9 (9.38)	87 (90.63)	0.54	
**Always defecate in a toilet**					
No	13	0	13 (100.00)		
Yes	306	31 (10.13)	275 (89.87)	0.62	
**Always keep your nails short**					
No	68	11 (16.18)	57 (83.82)		
Yes	251	20 (7.97)	231 (92.03)	0.047*	
**Biting or sucking fingers for play**					
No	241	22 (9.13)	219 (90.87)		
Yes	78	9 (11.54)	69 (88.46)	0.52	
**Washing hands before handling food**					
No	80	6 (7.50)	74 (92.50)		
Yes	239	25 (10.46)	214 (89.54)	0.52	
**Playing with pets such as dogs and cats**					
No	196	14 (7.14)	182 (92.86)		
Yes	123	17 (13.82)	106 (86.18)	0.05	
**Drinking water source**					
Filtered	290	28 (9.66)	262 (90.34)		
Unfiltered	29	3 (10.34)	26 (89.66)	0.75	

*Significant association based on Fisher’s Exact Testing.

STHs: Soil-transmitted helminth.

### Predictive spatial risk distribution map

A comparative analysis was conducted to evaluate the performance of three different model architectures using an augmented training dataset of publicly available datasets. A detailed comparison of performance metrics (Table 4) reveals that our customized CNN demonstrated the lowest performance among the evaluated models. In contrast, DenseNet121 emerged as the top-performing model, showing its superior capability in feature extraction and learning from the enriched data. This finding suggests that DenseNet121’s inherent architectural design, characterized by its densely connected layers, is particularly effective at capitalizing on the additional information provided by the augmented training dataset.

**Table 4:** Performance metrics of each classification model.

Model	Accuracy	Precision	Recall	F1-score
Our customized CNN	0.08	0.03	0.08	0.02
ResNet50	0.82	0.83	0.82	0.82
DenseNet121	0.89	0.91	0.89	0.87

**Fig. 4:**
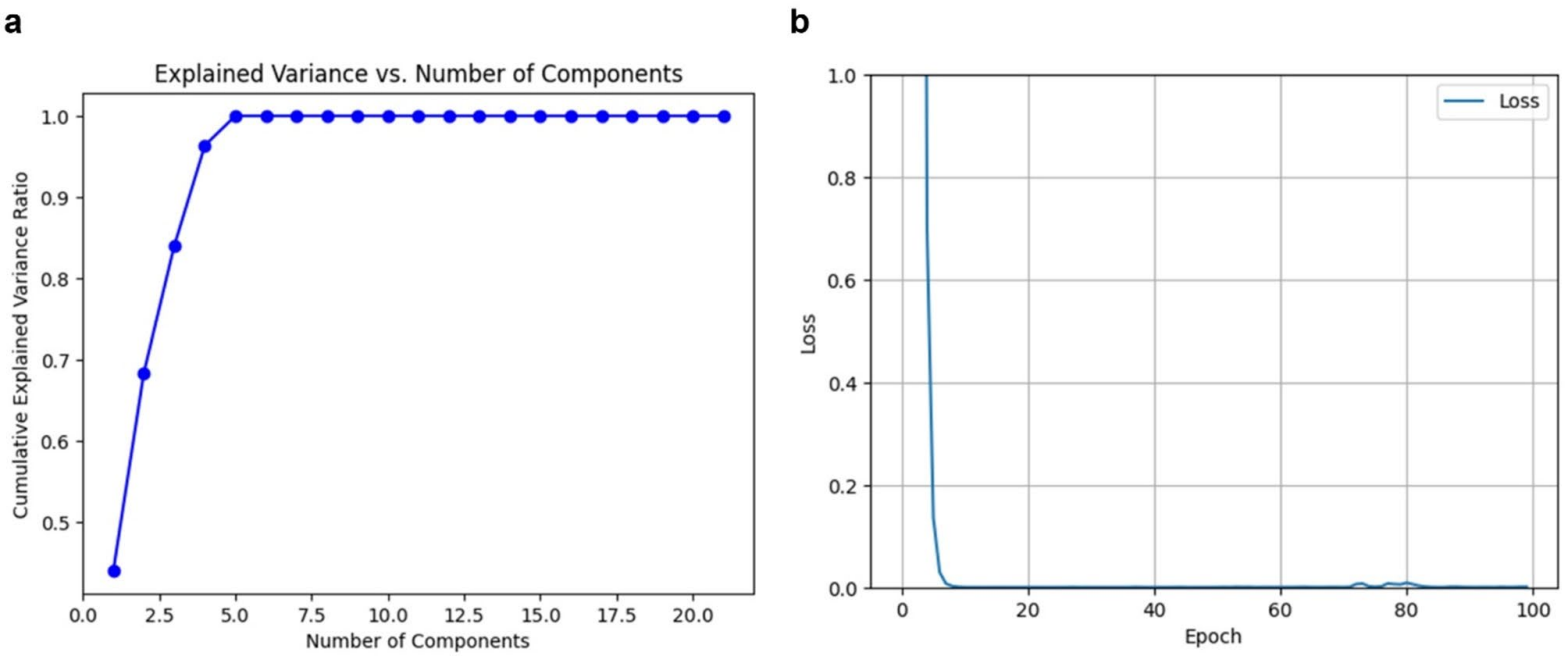
Dimensionality reduction and model training performance for STH infection risk prediction. (**a**) Cumulative explained variance ratio against number of principal components, showing that 4 components capture 95% of total variance. (**b**) Training loss curve of the ANN model demonstrating rapid convergence within 20 epochs.

**Fig. 5:**
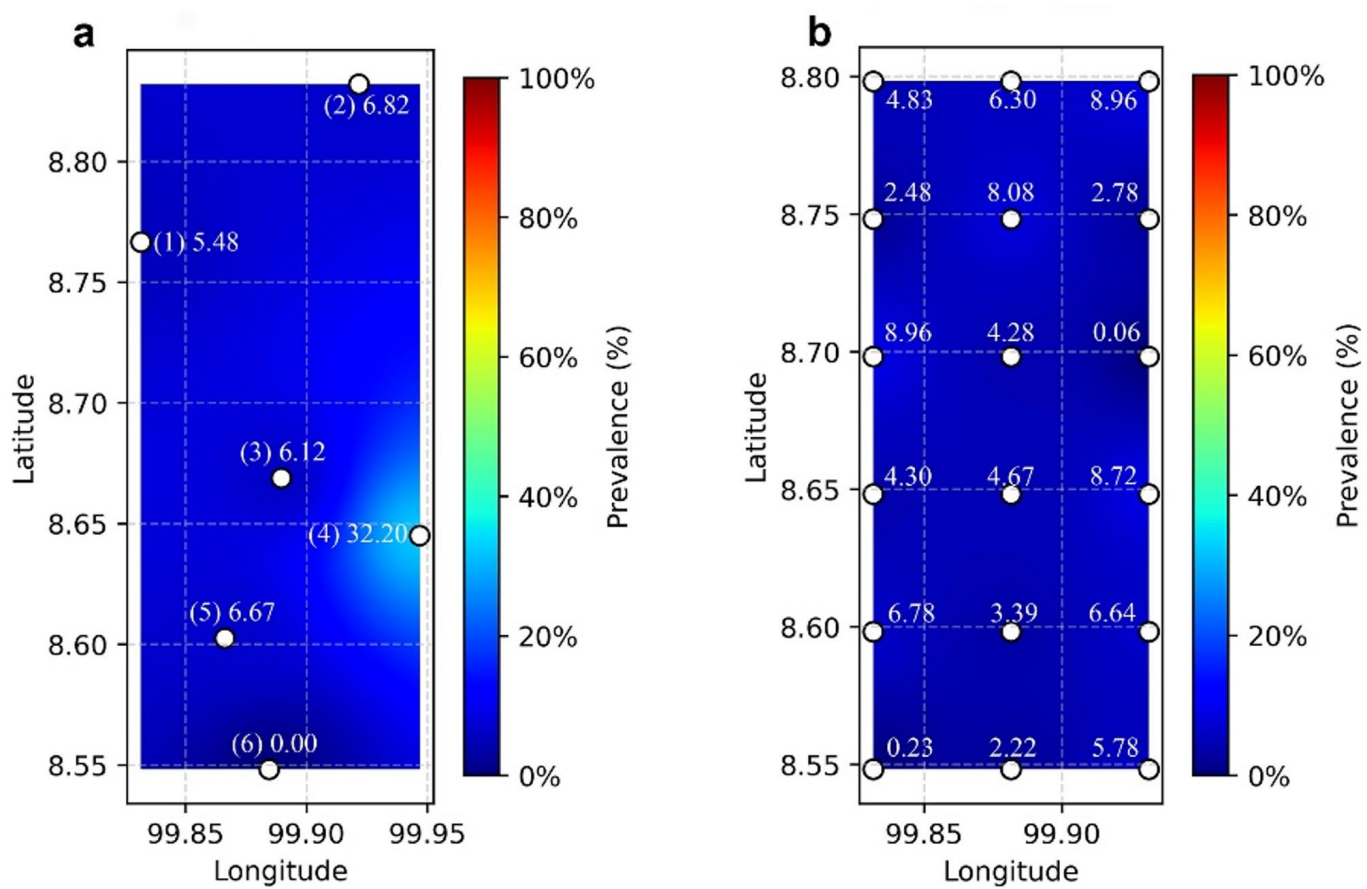
Comparative heat maps of STH prevalence with white circles indicating geographic locations on latitude-longitude coordinates. (**a**) Observed STH prevalence based on field data from six sub-districts in Thasala District: (1) Taling Chan, (2) Klai, (3) Thai Buri, (4) Thasala, (5) Moklan, and (6) Don Tako. (**b**) Predicted STH prevalence generated by the CNN-PCA-ANN model incorporating environmental and spatial features. The color gradient ranges from blue (low prevalence) to red (high prevalence), with the color bar showing prevalence percentages for direct comparison between observed and predicted patterns.

Principal component analysis was employed to reduce the dimensionality of the dataset from 21 parameters to 4 principal components, while retaining 95% of the total variance (Fig. 4a). This reduction not only streamlined the model complexity but also mitigated issues related to multicollinearity among input variables. Subsequently, an ANN was trained to regress the prevalence percentage, as evidenced by the training loss curve (Fig. 4b). The refinement of the model was further validated through testing, with the total sum-square error calculated as 4.06e-4, highlighting the model’s precision and effectiveness in prediction. This integrated PCA-ANN methodology proves effective for processing high-dimensional environmental data while maintaining robust predictive performance in estimating STH infection prevalence. To assess the spatial accuracy of our model’s predictions, a comparative visualization of STH prevalence was generated (Fig. 5). The heat map derived from empirical data collected in rural school settings provides direct evidence of localized infection patterns (Fig. 5a). When applied to the study area, our predictive model effectively replicates these observed spatial distributions (Fig. 5b), further validating the model’s accuracy in estimating prevalence while enabling identification of high-risk areas.

## Discussion

Our study explored the prevalence, associated risk factors, and predictive spatial risk distribution map of STH infections among school-aged children in Thasala District, Nakhon Si Thammarat Province, Thailand. This represents the first such report from southern Thailand in five years. With an overall prevalence of 9.72%, the findings confirm that STH infections remain a public health concern in rural southern Thailand, especially among children who are more vulnerable due to frequent exposure to contaminated soil and poor hygiene practices. The most detected parasite was *T. trichiura*, followed by hookworm. This infection pattern contrasts with previous studies in the southern region, which identified hookworm as the dominant species^8,10^. The success of previous deworming programs targeting hookworms but potentially less effective against *T. trichiura* might explain this shift in species predominance. Furthermore, the detection methods employed in this study may have enhanced sensitivity for *T. trichiura* compared to previous investigations in the region^23^. These findings demonstrate the dynamic nature of STH epidemiology and highlight the need for time- and location-specific assessments to develop effective control strategies. Interestingly, among the six sub-districts examined in this study, Thasala sub-district exhibited the highest prevalence of STH infections, with *T. trichiura* being the predominant species detected. The elevated prevalence of *T. trichiura* in this area may be attributed to several interconnected factors. First, parasite-related factors include the reduced efficacy of albendazole against *T. trichiura* compared to other STH species^24^which may contribute to persistent infections in areas where this anthelmintic has been previously administered as part of deworming programs. Second, environmental factors such as the forested and humid characteristics of the Thasala area may provide optimal conditions for the survival and development of *T. trichiura* infective stages, where the combination of adequate moisture, shade, and suitable soil conditions creates an environment conducive to parasite persistence and transmission. Third, host behavioral factors may include behavioral patterns specific to children in this area, such as frequent play activities in shaded, moisture-rich environments where *T. trichiura* eggs can survive and mature. Behavioral analysis revealed that most hygiene-related practices, including handwashing, food hygiene, and wearing shoes, were not significantly associated with STH infections in this study. While this contrasts with findings from several studies that underscore the importance of hand hygiene and safe food practices^25–27^the discrepancy may reflect cultural differences in hygiene routines, reporting bias in self-administered questionnaires, or the relatively small sample size, which limits statistical power. Nonetheless, the study did identify one significant behavioral risk factor: not cutting nails was associated with a markedly higher infection rate. This supports earlier findings that personal hygiene, particularly nail care, and plays a critical role in interrupting fecal-oral transmission pathways^28^. Additionally, playing with pets was associated with higher infection prevalence. Although not conclusive, this suggests that zoonotic transmission or exposure to contaminated environments via animal contact could contribute to hookworm risk in rural communities. Educational campaigns focused on hygiene and sanitation, as well as routine deworming programs, could further mitigate infection risks, as advocated by WHO guidelines on STH control in school-aged children^29^. Our study developed an innovative predictive model for STH infection risk by integrating CNN-based satellite imagery classification with ANN analysis after PCA dimensionality reduction. Using the Land-Use Scene Classification database, we found that DenseNet121 significantly outperformed traditional CNN architectures with augmented training data. Its densely connected design proved particularly effective at extracting features from enriched datasets^30^. Satellite images from the six schools were standardized to 256 × 256 pixels, effectively balancing structural detail with computational efficiency. This standardization approach facilitated robust comparative analyses across diverse school environments^31^. Reducing the 21-parameter dataset to four principal components via PCA, while retaining 95% of the variance, effectively addressed multicollinearity and streamlined the subsequent regression analysis. The low sum-square error observed during ANN testing demonstrates the efficacy of this PCA-ANN approach in predictive modeling^32^. This integrated analytic framework enabled the identification of specific environmental features, particularly higher proportions of agricultural land and closer proximity to water bodies, that were positively associated with elevated STH prevalence. The strong correlation between predicted and observed patterns validates our methodology’s practical utility for targeted public health interventions.

Our study has certain limitations. First, the use of single-day stool sample collection may have reduced diagnostic sensitivity, likely leading to an underestimation of the true prevalence of STHs infections. Collecting samples over multiple days would improve detection accuracy^33^. Second, the relatively small sample size may limit the generalizability of the findings. Additionally, the analysis did not separate risk factors by individual STH species, despite their differing modes of transmission. Future studies should consider species-specific analyses to better understand the distinct behavioral and environmental factors associated with each type of infection. A larger sample in future studies would enhance the statistical power of the results. Third, while our approach successfully generates spatial risk distribution maps, translating findings into targeted interventions remains challenging. Future work should implement back-analysis^34^SHAP-based feature importance^35^and sensitivity analysis^36^ to identify key environmental factors associated with STH prevalence. Model validation through targeted seasonal parasitological surveys in predicted high-risk areas is also essential. Furthermore, integrating GIS with our CNN-PCA-ANN approach would enhance our methodology by enabling real-time data collection, improving prediction accuracy, and facilitating locally-tailored interventions.

## Conclusion

This study confirms STH infections remain a public health challenge among schoolchildren in rural southern Thailand, with an overall prevalence of 9.72%. *Trichuris trichiura* and hookworm were the predominant parasites, with mono-infections occurring more frequently than mixed infections. Poor hygiene practices, particularly not cutting nails, were significantly associated with infection risk. Our innovative predictive model integrating CNN-based land-use classification with ANN processing of PCA-refined data generated precise spatial risk distribution maps for STH infection. Comparative analyses between empirical heat maps and model outputs validated this approach. These findings demonstrate the effectiveness of combining satellite imagery analysis with machine learning for targeting public health interventions. To reduce STH burden, strengthening school-based hygiene education, improving sanitation, and implementing environmental control strategies guided by this geographic data will be essential.

## Supplementary Information

Below is the link to the electronic supplementary material.

Supplementary Material 1

## Data Availability

Data is provided within the supplementary information file.
